# Estimating effect of intervention delivery in the evaluation of complex intervention with various levels of compliance

**DOI:** 10.1186/1745-6215-14-S1-O89

**Published:** 2013-11-29

**Authors:** Baptiste Leurent, Mike Crawford, Kalaitzaki Eleftheria, Irwin Nazareth

**Affiliations:** 1 UCL PRIMENT CTU, London, UK; 2 UCL Department of Primary Care and Population Health, London, UK; 3 Centre for Mental Health, Imperial College, London, UK; 4 Institute of Cancer Research, Sutton, UK

## 

The evaluation of complex intervention with a randomised trial is often undermined by varying levels of participant compliance to the intervention. It is important to evaluate the causal effect of different levels of the intervention to gain a fuller understanding of the effectiveness of the intervention when complied with. For example, when evaluating a psychotherapeutic intervention, the effect of attending a session, in addition to the intention-to-treat effect of being offered the therapy, is valuable. Instrumental variables (IV) approach can be used for the estimation of such effect, by handling confounders usually associated with compliance. IV relies on the use of an "instrument", related to the dependent variable only through its effect on the independent variable. Although instrumental variables have been commonly used in econometrics and social sciences, they are seldom applied to the evaluation of complex health intervention. In this presentation, we will demonstrate how the randomisation can be used as an instrument to determine the causal effect of the intervention. We will discuss the theory and assumptions underpinning this approach, and the scope for applying this method to trials on complex intervention. This will be illustrated through a large three-arm randomised controlled trial of art therapy for schizophrenia (MATISSE) where attendance at the intervention sessions was low, and non-compliance was an important limitation for interpreting trial results. The use of instrumental variable techniques enabled a fuller understanding of the effect of the intervention.

